# Liposomal Bupivacaine Versus Bupivacaine and Dexamethasone Intercostal Nerve Blocks for Robotic Thoracic Surgery: A Randomized Clinical Trial

**DOI:** 10.7759/cureus.62085

**Published:** 2024-06-10

**Authors:** Kingsuk Ganguly, Noud Van Helmond, Adam Friedman, Rabeel Ahmad, Frank Bowen, David D Shersher, Ludmil V Mitrev

**Affiliations:** 1 Department of Anesthesiology, Cooper University Hospital, Camden, USA; 2 Department of Anesthesiology, Cooper Medical School of Rowan University, Camden, USA; 3 Department of Anesthesiology, Rowan-Virtua School of Osteopathic Medicine, Stratford, USA; 4 Department of Cardiothoracic Surgery, Cooper University Hospital, Camden, USA; 5 Department of Thoracic Surgery, Cooper University Hospital, Camden, USA

**Keywords:** exparel, bupivacaine, liposomal bupivacaine, robotic thoracic surgery, acute postoperative pain, intercostal nerve block, regional anesthesia, adjuvant dexamethasone

## Abstract

Introduction

For peripheral nerve blocks, using either the liposomal formulation of bupivacaine or plain bupivacaine with epinephrine and dexamethasone as an adjuvant has been shown to improve postoperative pain scores. In a single-blinded, randomized controlled study of patients undergoing robotic-assisted thoracoscopic surgery, we determined if bupivacaine with epinephrine and dexamethasone was noninferior to liposomal bupivacaine mixed with plain bupivacaine when administered intraoperatively as an intercostal nerve block (INB).

Methods

A total of 34 patients undergoing robotic-assisted thoracoscopic surgery were randomized to receive one of two injectate mixtures during their intraoperative INB. Group LB was administered 266 mg of 13.3 mg/mL liposomal bupivacaine with 24 mL of 0.5% plain bupivacaine, while Group BD was given 42 mL of 0.5% bupivacaine with epinephrine and 8 mg of dexamethasone. The primary outcomes were mean postoperative numerical pain ratings and mean postoperative opioid analgesic requirements. Secondary outcomes included adjuvant pain medication consumption, hospital length of stay, and total opioid use in oral morphine equivalents.

Results

Group LB exhibited no significant difference in pain scores (p = 0.437) and opioid analgesic requirement (p = 0.095) within the 72-hour postoperative period when compared to Group BD. The median total postoperative opioid requirement was 90 mg in Group LB, compared to 45 mg in Group BD. There were no significant differences in the use of postoperative adjuvant pain medications (gabapentin, p = 0.833; acetaminophen, p = 0.190; ketorolac, p = 0.699). Hospital length of stay did not differ between the groups.

Conclusions

INBs with the addition of dexamethasone as an adjuvant to 0.5% bupivacaine with epinephrine provided noninferior postoperative analgesia compared to liposomal bupivacaine mixed with plain 0.5% bupivacaine.

## Introduction

Postoperative pain associated with thoracic surgery has been successfully managed with intercostal nerve blocks (INBs), thoracic epidural analgesia, and paravertebral blocks. In particular, studies have shown that INBs reduce postoperative pain and opioid requirements when compared to patients who underwent surgery without regional anesthesia and were solely administered systemic analgesia [1]. Results from a meta-analysis concluded that INBs were clinically noninferior to thoracic epidural analgesia and paravertebral blocks in patients who underwent thoracic surgery [2].

Bupivacaine is a commonly used and cost-effective local anesthetic administered in peripheral nerve blocks, and its efficacy in providing postoperative analgesia has been well-characterized [3-5]. A common technique in regional anesthesia is to use bupivacaine with epinephrine due to its known effect on local vasoconstriction, which prevents systemic reabsorption of local anesthetic, thereby maximizing tissue concentrations and increasing its duration of action [6]. Without epinephrine, therapeutic dosing of bupivacaine should not exceed 2-2.5 mg/kg in order to avoid symptoms of local anesthetic systemic toxicity [7,8]. When epinephrine is added, this value increases to 3 mg/kg [7,8].

A liposomal formulation of bupivacaine, marketed as Exparel (Pacira, Inc., USA), allows for a slower release of bupivacaine to provide a longer duration of action [9]. It is currently approved as a single-dose infiltration with a maximum recommended dose of 266 mg, though the FDA prescribing information describes 10 randomized, controlled trials that used Exparel doses ranging from 66 mg to 532 mg [10]. Liposomal bupivacaine cannot be admixed with other local anesthetics except for plain bupivacaine, which should not exceed a 1:2 ratio of plain bupivacaine to liposomal bupivacaine [10]. Currently, liposomal bupivacaine is FDA-approved for use in interscalene brachial plexus nerve blocks, sciatic nerve blocks in the popliteal fossa, and adductor canal blocks; however, further evidence suggests that liposomal bupivacaine also reduces the need for analgesic medications following arthroscopic knee surgery, relative to the standard unencapsulated local anesthetics administered in peripheral nerve blocks [9,11]. When used during thoracic surgeries, liposomal bupivacaine provided significantly better postoperative pain control and shorter hospital lengths of stay compared to using standard bupivacaine alone [12,13].

Another method of prolonging the duration and increasing the efficacy of local anesthetics is by adding an adjuvant medication, such as dexamethasone [14]. Perineural dexamethasone, given as an adjuvant for peripheral nerve blocks, has been associated with a faster onset of anesthesia, longer duration of anesthesia/analgesia, decreased postoperative pain intensity and decreased postoperative analgesia requirements compared with local anesthetic alone [15]. Adjuvant dexamethasone has also been successfully utilized to reduce postoperative pain and promote earlier recovery in patients when used as an intraoperative local infiltration with local anesthetics during knee arthroplasty [16].

Current evidence is limited to support the equivalence of using either liposomal bupivacaine or plain bupivacaine with dexamethasone as an adjuvant medication when administering INBs. Both plain bupivacaine and dexamethasone are considered cost-effective medications that have the potential to provide the same quality of care when compared to liposomal bupivacaine. Therefore, we conducted a prospective, single-blinded, randomized controlled study aimed at determining whether the addition of dexamethasone to bupivacaine with epinephrine during INBs for robotic-assisted thoracic surgery is noninferior to INBs with liposomal bupivacaine based on postoperative pain scores and postoperative analgesic requirements.

## Materials and methods

Study design

This was an investigator-initiated, prospective, single-center, single-blinded noninferiority trial conducted at a tertiary academic hospital (Cooper University Hospital, Camden). This study was approved by the hospital’s Institutional Review Board. The recommendations set forth by the Consolidated Standards of Reporting Trials were followed in the reporting of the results of this clinical trial. Data analysis was performed once patient enrollment was completed. Patients aged 18-90 who were scheduled for robotic-assisted thoracic surgery, specifically wedge resection and lobectomy, were considered eligible to participate in this study. Patients who underwent conversion to thoracotomy incision were included for analysis per the intention to treat principle. Patients were considered ineligible if they had any chronic pain syndrome, active or prior intravenous drug use, chronic oral steroid use, a history of opioid abuse/use disorder, and liver failure. Additional exclusion criteria included emergent thoracic surgical cases, patients weighing less than 70 kg, pregnant women, incarcerated patients, and uninsured patients. Those who had a history of allergic reaction to any medications used in this study (bupivacaine, dexamethasone, liposomal bupivacaine, epinephrine), as well as those who were non-verbal or could not rate their pain on a numerical rating scale, were also excluded.

Randomization and blinding

After obtaining written informed consent, patients who met inclusion criteria were enrolled and randomized using a computer-generated randomization table in a 1:1 sequential fashion, either to the plain bupivacaine with epinephrine and adjuvant dexamethasone (BD) group, or the liposomal bupivacaine (LB) group. The random allocation sequence was performed using the random number generator tool in Microsoft Excel (Microsoft Corporation Inc., Redmond, WA, USA). Patients were randomized into two groups consisting of 17 patients each. A double-blinded design was not feasible because the formulation of liposomal bupivacaine is cloudy in appearance, whereas plain bupivacaine and dexamethasone are both clear solutions. Thus, the study staff and the surgeon were not blinded. All patients, however, were blinded as to their study group allocation.

Sample size determination

In order to detect a two-point pain score difference between the groups (non-inferiority margin), a minimum sample size of 17 per group was required based on a standard deviation of 2, a p-value of 0.05 (two-tailed) and 80% power. Sample size calculation was performed using PASS (NCSS LLC, Kaysville, UT, USA).

Interventions

Both groups received INBs and wound infiltration at the surgical sites. The injections were performed intraoperatively by the surgeon. All participating physician administrators are board-certified in thoracic surgery and are equally skilled in performing this anatomical block. Group LB was administered 266 mg of liposomal bupivacaine (13.3 mg/mL) mixed with 24 mL of 0.5% plain bupivacaine. Group BD was given 42 mL of 0.5% bupivacaine with epinephrine and 8 mg (2 mL) of dexamethasone. The total volume of injectate in both groups was 44 mL and was split evenly (22 mL each) between the INB and the wound infiltration of subcutaneous tissue.

Outcomes

The first primary outcome measure in this study was average postoperative pain scores on an 11-point numerical rating scale (NRS) at 1, 6, 12, 24, 36, 48, and 72 hours after surgery. The second was mean postoperative opioid requirement over the same 72-hour period, where measurements were converted to Oral Morphine Equivalents given that patients were administered various opioids during the postoperative period, most commonly oxycodone and hydromorphone. Additional outcome measurements included total opioid requirement over 72 hours postoperatively, hospital length of stay, length of stay in the post-anesthesia care unit (PACU), and average use of adjuvant pain medications (gabapentin, ketorolac, and acetaminophen).

Statistical analysis

The management, assessment, and analysis of the data were carried out in a blinded fashion. Demographic and clinical characteristics (i.e., age, gender, comorbidities) are presented with the number of subjects and percentages, as well as means with standard deviations. Prior to analysis, the primary and secondary outcome data were tested for normality using the Shapiro-Wilk test. Between-group differences were assessed to determine the degree of similarity between subjects in the two groups using a t-test for continuous variables and Fisher’s exact test for categorical variables. The primary outcomes, mean NRS pain scores and OME requirements, were analyzed as continuous variables. A noninferiority margin of 2 points at 24 hours on the NRS was considered clinically equivalent. Noninferiority was established if the noninferiority margin (2 points) fell within the upper bound of the 95% confidence interval of the difference between the two groups. If the data did not follow a normal distribution, then a normalized rank transformation was applied to the data prior to analysis. Group differences at different time points were tested using ANCOVA for repeated measures with demographic and clinical variables used as covariates in a linear model. A Bonferroni correction was used to adjust for multiple comparisons. A p-value of 0.05 (two-tailed) was used to determine statistical significance. The program used for statistical analysis was IBM SPSS Statistics for Windows, Version 29 (Released 2023; IBM Corp., Armonk, New York, United States).

## Results

**Figure 1:**
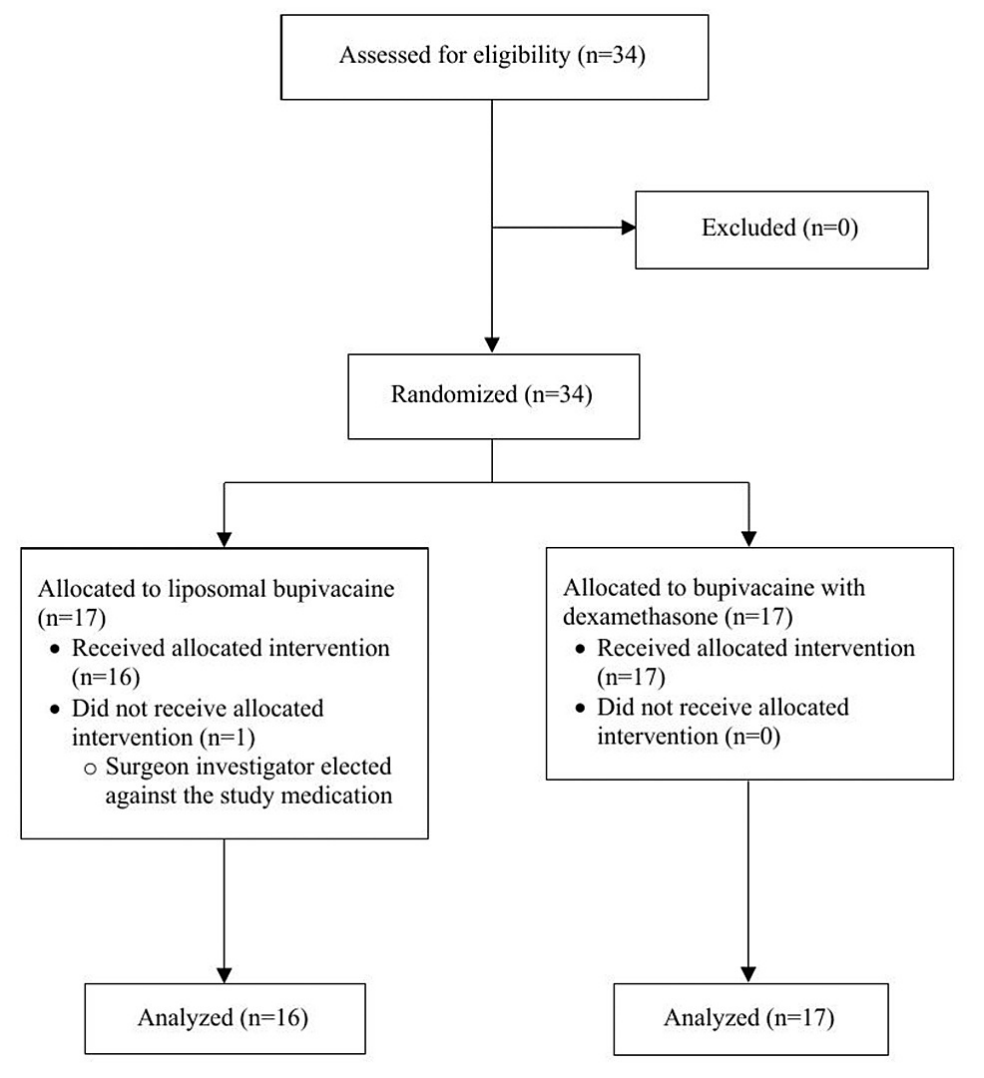
Consolidated Standards for Reporting Trials (CONSORT) Flow Diagram

A total of 34 patients were enrolled and randomized between February 7, 2019 and March 31, 2022 into two distinct treatment groups, consisting of 17 patients each. In the liposomal bupivacaine group (Group LB), all patients except for one received the study medication and were included in the final analysis. One patient did not receive the study medication for surgical reasons and was removed from analysis. All patients randomized to the bupivacaine with dexamethasone group (Group BD) received the intervention and were analyzed. Five patients underwent intraoperative conversion to open thoracotomy, two in Group LB and three in Group BD, all of which were included in the analysis. Therefore, 33 patients completed the study (Group LB: n = 16; Group BD: n = 17). The clinical trial period ended once all postoperative data was collected. There were no reported harms or unintended effects that pertained to the study interventions. The Consolidated Standards of Reporting Trials flow diagram presented in Figure 1 details the progress of patient participation throughout the study.

Table 1 displays the patient demographics and procedure duration for both groups. Secondary outcomes, which included PACU length of stay, hospital length of stay, and total OME requirement over the 72-hour postoperative period, were found to be statistically similar between groups (Table 2).

**Table 1:** Patient Characteristics and Procedure Duration LB, liposomal bupivacaine; BD, bupivacaine with dexamethasone; IQR, interquartile range; BMI, body mass index; SD, standard deviation

Category	Group LB (n=16)	Group BD (n=17)
Age in years, median (IQR)	69 (63 – 72)	71 (64 – 74)
BMI in kg/m^2^, mean (SD)	28 (5)	30 (5)
Sex, female, n (%)	8 (50)	3 (18)
Procedure duration in minutes, median (IQR)	189 (138 – 245)	269 (188 – 331)

**Table 2:** Secondary Outcomes IQR, interquartile range; LB, liposomal bupivacaine; BD, bupivacaine with dexamethasone; PACU, post-anesthesia care unit; LOS, length of stay; OME, oral morphine equivalent

Category, median (IQR)	Group LB	Group BD	p-value
PACU LOS in minutes	527 (346 – 1351)	347 (186 – 539)	0.084
Hospital LOS in days	5 (3 – 6)	5 (3 – 6)	0.798
Total OME requirement in mg	90 (23 – 170)	45 (21 – 93)	0.182

**Figure 2:**
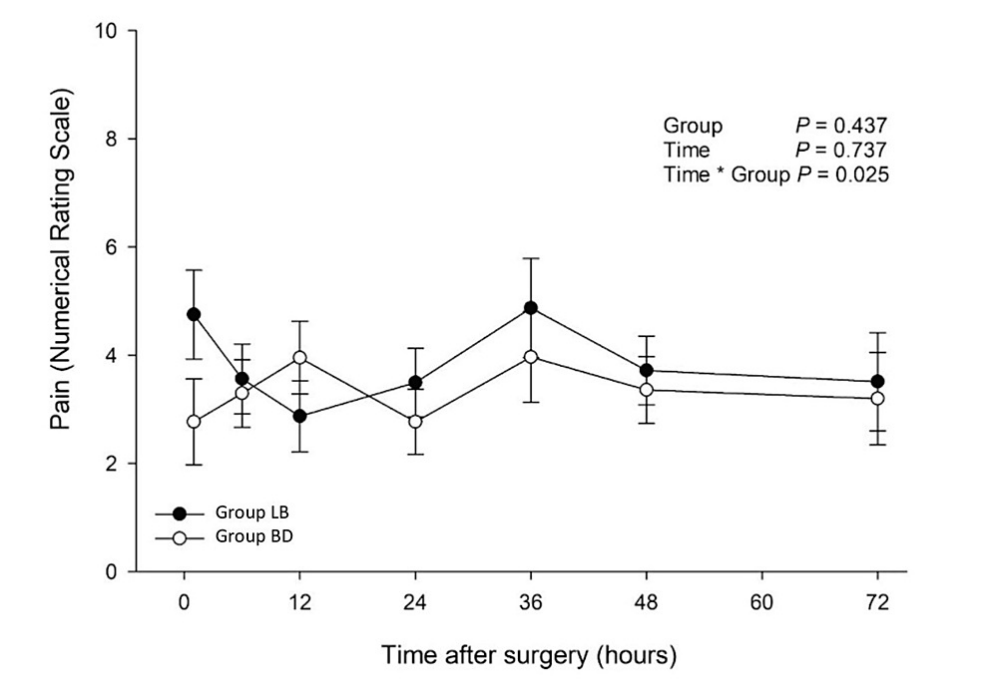
Numerical Pain Scores in the 72-Hour Postoperative Period LB, liposomal bupivacaine; BD, bupivacaine with dexamethasone.

The average pain scores in Group LB and Group BD over the 72-hour postoperative period are shown in Figure 2. No significant differences were found when groups were analyzed independent of time (p = 0.437). No significant difference in NRS pain scores was appreciated on post hoc analysis on each postoperative day.

**Figure 3:**
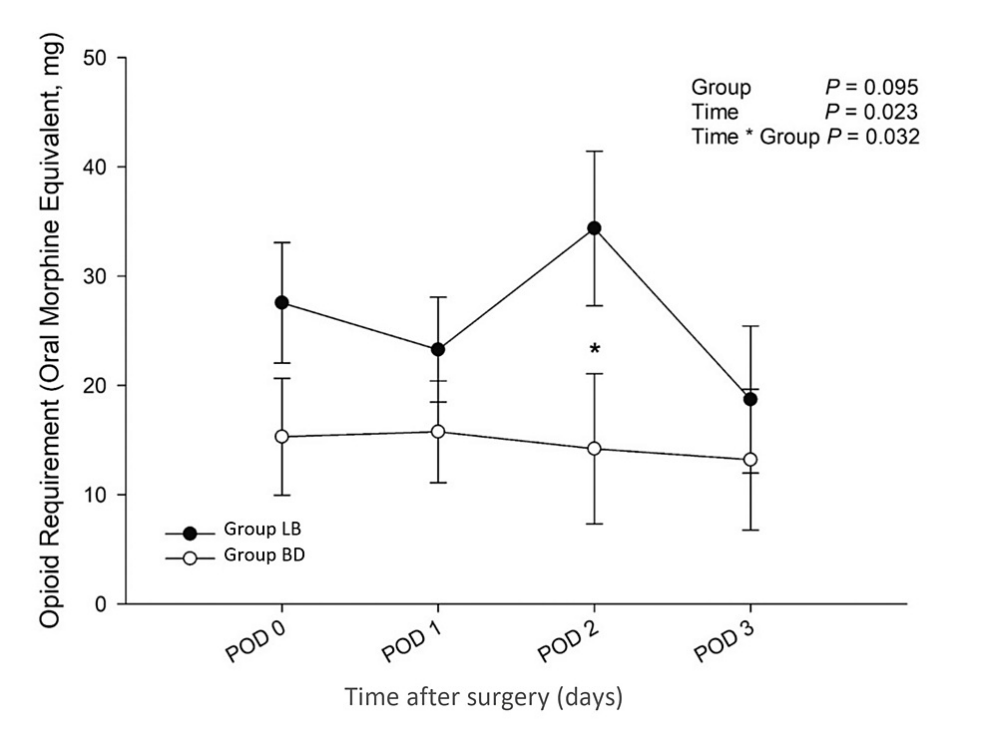
Opioid Requirement in the 72-Hour Postoperative Period LB, liposomal bupivacaine; BD, bupivacaine with dexamethasone; POD, postoperative day.

Mean postoperative opioid requirements across the 72-hour postoperative period were not found to be significantly different between groups (p = 0.095); however, they were found to be significantly lower in Group BD compared to Group LB when time was considered as a covariate (time*group interaction, p = 0.032, Figure 3). When stratified by the individual postoperative days, Group BD had a significantly lower analgesic requirement on postoperative day 2 relative to Group LB (*p = 0.049).

**Figure 4:**
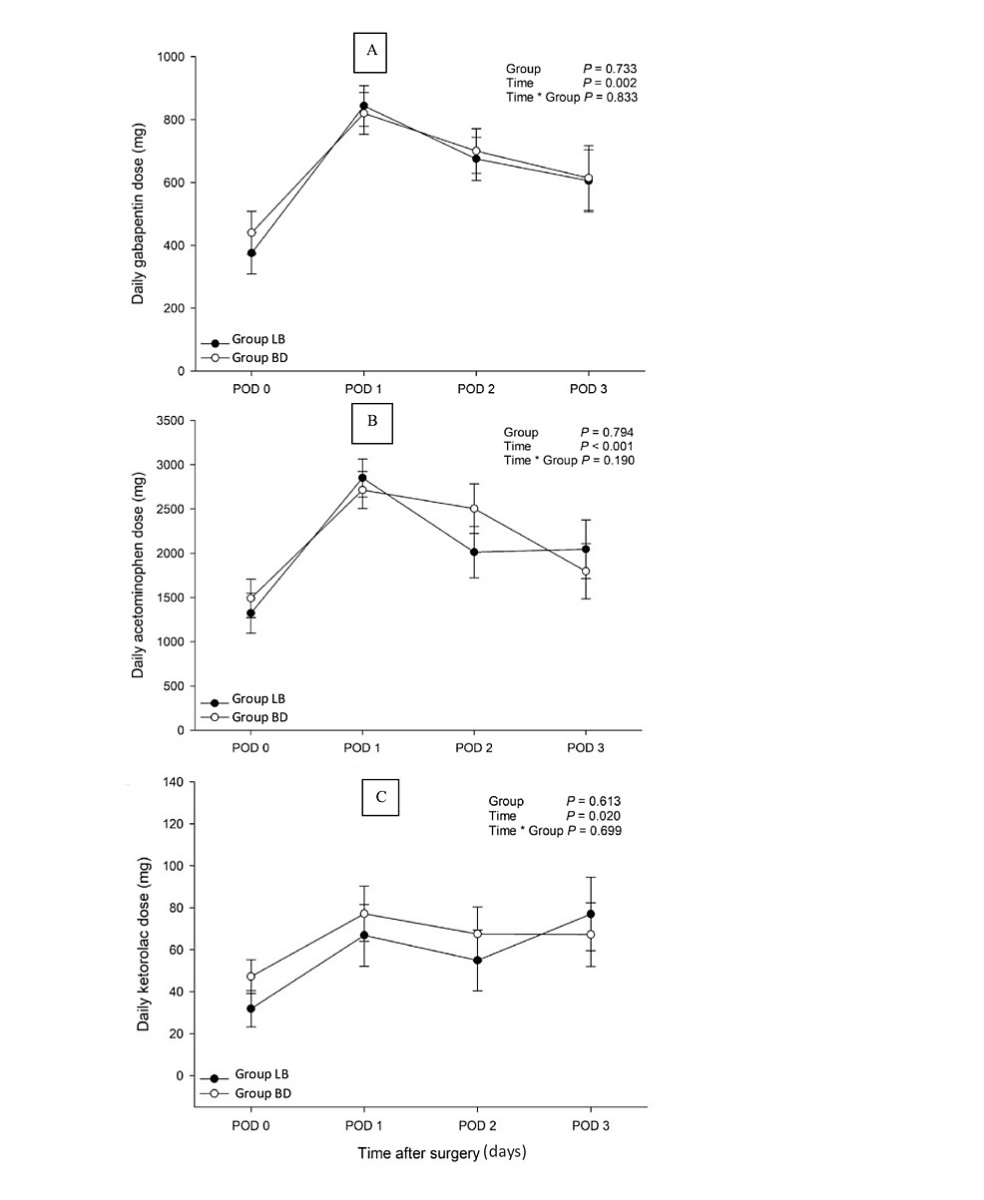
Use of Adjuvant Pain Medications Within Three Days After Surgery A, Panel A use of adjuvant analgesic gabapentin within three days after surgery; B, Panel B use of adjuvant analgesic acetaminophen within three days after surgery; C, Panel C use of adjuvant analgesic ketorolac within three days after surgery; LB, liposomal bupivacaine; BD, bupivacaine with dexamethasone; POD, postoperative day.

The adjuvant pain medications that were selected for analysis were based on whether or not greater than 25% of the patients in each group used the medication over the 72-hour postoperative period. Figure 4 outlines the mean consumption of gabapentin, acetaminophen, and ketorolac of both groups over this period. No significant between-group differences were observed for all three adjuvant pain medications: gabapentin (p = 0.833), acetaminophen (p = 0.190), and ketorolac (p = 0.699).

## Discussion

Our study suggests that the use of bupivacaine with adjuvant dexamethasone for INBs and wound infiltration in patients undergoing robotic-assisted thoracoscopic surgery is noninferior to the use of liposomal bupivacaine. Patients who received INBs with bupivacaine and dexamethasone experienced statistically equivalent postoperative pain scores and opioid analgesic requirements when compared to those who received INBs with liposomal bupivacaine. Though not statistically significant, patients in the BD group consumed, on average, half the total amount of opioids in the 72-hour postoperative period compared to those in the LB group.

Perioperative characteristics like PACU length of stay and hospital length of stay were not significantly different between the two groups, implying that the postoperative time course was more likely a reflection of the type of surgery that the groups underwent. There were also no significant differences between groups with regard to taking adjuvant pain medications like gabapentin, acetaminophen, and ketorolac. This suggests that using these medications in the postoperative period did not have a significant impact on the opioid requirement and the pain scores observed in the treatment groups.

Dexamethasone stands to be a cost-effective and clinically beneficial adjuvant medication for INBs, and additional improvements in patient outcomes have been characterized in recent literature. Proposed mechanisms by which corticosteroids can potentiate the effect of local anesthetics include modulation of neuronal potassium channels, reduction of blood flow in neuronal vasculature, and inhibition of nociceptive pain receptors in peripheral nerve fibers [14,17,18]. Vasconcelos et al. concluded that perineural dexamethasone reduced pain intensity and patient requirement for rescue analgesia during the postoperative period when combined with levobupivacaine in an interscalene brachial plexus block for shoulder arthroscopic surgery [19]. In patients who underwent an open reduction and internal fixation for upper extremity fracture, perineural administration of dexamethasone with ropivacaine reduced rebound pain compared to patients who received ropivacaine alone [20]. However, there has only been one randomized clinical trial that similarly compared two treatment groups receiving either liposomal bupivacaine or plain bupivacaine with dexamethasone, studied in the context of interscalene brachial plexus blocks for shoulder surgery [21]. Kim et al. concluded that postoperative pain scores were non-inferior in both treatment groups; however, their study outcomes are currently disputed [21-23]. Considering the controversial findings in these reports, as well as the evidence provided by our study, further investigations with larger study populations are required to assess the clinical utility of dexamethasone as an adjuvant for peripheral nerve blocks.

There are several limitations to consider with this study. First, the cloudy appearance of liposomal bupivacaine is visually distinct from the clear formulations of standard bupivacaine and dexamethasone, preventing this study from being double-blinded. Next, this study did not control the use of intravenous dexamethasone perioperatively, which would have allowed us to determine if the benefits observed with adjuvant dexamethasone were from perineural action or systemic absorption. Eighteen out of the 33 enrolled patients received low-dose (4 mg) IV dexamethasone intraoperatively for prophylaxis of postoperative nausea and vomiting. In addition, it is likely that the patients whose procedures were converted to open thoracotomy may have experienced more pain relative to those whose procedures went as planned. This may have affected the significance observed in this study with regard to these nerve blocks and the clinical impact of the local anesthetics that were of interest in this study. Furthermore, this study did not examine the duration between INB administration and the patient's first request for postoperative opioid analgesia. This would have provided insight into whether adjuvant dexamethasone prolonged the patients’ postoperative narcotic-free period, in addition to reducing the total opioid requirement. Lastly, the use of dexamethasone for peripheral nerve blocks is considered off-label, despite its clinical efficacy reported in the literature.

## Conclusions

Patients who underwent robotic-assisted thoracoscopic surgery experienced similar postoperative pain and opioid consumption following their surgery when they received INBs with either plain bupivacaine with epinephrine and dexamethasone or liposomal bupivacaine mixed with plain bupivacaine. Perineural dexamethasone is an effective and cost-efficient medication and deserves greater consideration as an adjuvant to peripheral nerve blocks. We recommend further studies to compare variable doses of adjuvant dexamethasone used in INBs in order to achieve optimal postoperative anesthesia and analgesia, as well as the use of other adjuvant medications that can potentially increase the efficacy of peripheral nerve blocks.
